# Successful Treatment of Bleeding Gastric Varices with Splenectomy in a Patient with Splenic, Portal, and Mesenteric Thromboses

**DOI:** 10.1155/2013/273531

**Published:** 2013-09-03

**Authors:** Lior Menasherian-Yaccobe, Nathan T. Jaqua, Patrick Kenny

**Affiliations:** ^1^Department of Internal Medicine, Tripler Army Medical Center, 1 Jarrett White Road, Honolulu, HI 96859, USA; ^2^Gastroenterology Service, Tripler Army Medical Center, USA

## Abstract

A 59-year-old female with a history of multiple splanchnic and portal thromboses treated with warfarin underwent an esophagogastroduodenoscopy for cancer screening, and a polypoid mass was biopsied. One week later, she was admitted with upper gastrointestinal hemorrhage. Her therapeutic coagulopathy was reversed with fresh frozen plasma, and she was transfused with packed red blood cells. An esophagogastroduodenoscopy demonstrated an erosion of a gastric varix without evidence of recent bleeding. Conservative measures failed, and she continued to bleed during her stay. She was not considered a candidate for a shunt procedure; therefore, a splenectomy was performed. Postoperative esophagogastroduodenoscopy demonstrated near complete resolution of gastric varices. One year after discharge on warfarin, there has been no recurrence of hemorrhage. Gastric varices often arise from either portal hypertension or splenic vein thrombosis. Treatment of gastric variceal hemorrhage can be challenging. Transjugular intrahepatic portosystemic shunt is often effective for emergency control in varices secondary to portal hypertension. Splenectomy is the treatment for varices that arise from splenic vein thrombosis. However, treatment of gastric variceal hemorrhage in the context of multiple splanchnic and portal vein thromboses is more complicated. We report splenectomy as a successful treatment of gastric varices in a patient with multiple extrahepatic thromboses.

## 1. Introduction

Gastric varices are less common than esophageal varices in patients with portal hypertension, occurring in up to 33% of patients [1–3]. Gastric varices are more common in patients with noncirrhotic portal hypertension and extrahepatic portal vein thrombosis, are associated with a lower incidence of bleeding, and have a higher mortality rate than esophageal varices [1–3]. Optimal management of gastric variceal bleeding is debatable, because of lack of data from large randomized controlled trials [3]. We present a case of gastric variceal bleeding caused by prehepatic venous thrombosis from essential thrombocythemia that was successfully treated with therapeutic splenectomy. 

## 2. Case Report

**Figure 1:**
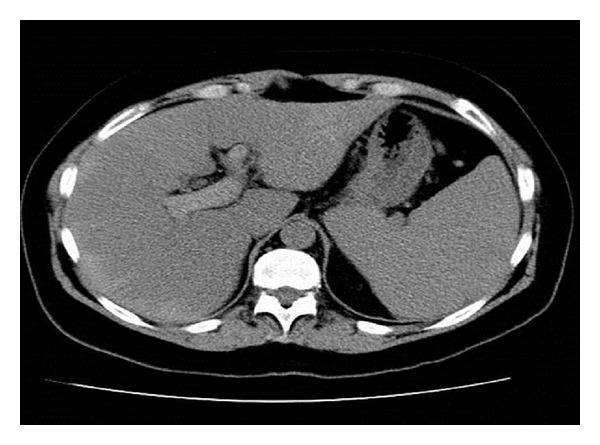
CT demonstrating portal thrombosis.

**Figure 2:**
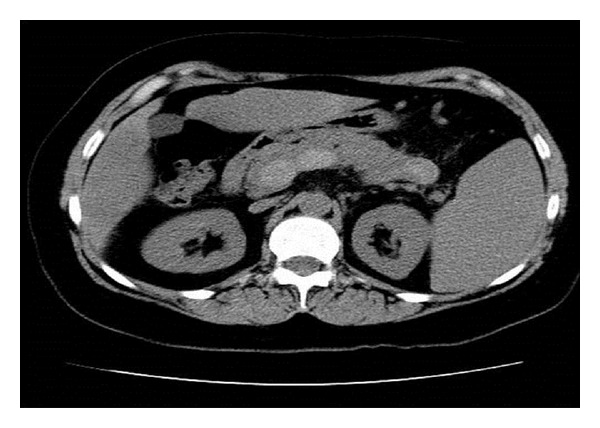
CT demonstrating splenic thrombosis.

**Figure 3:**
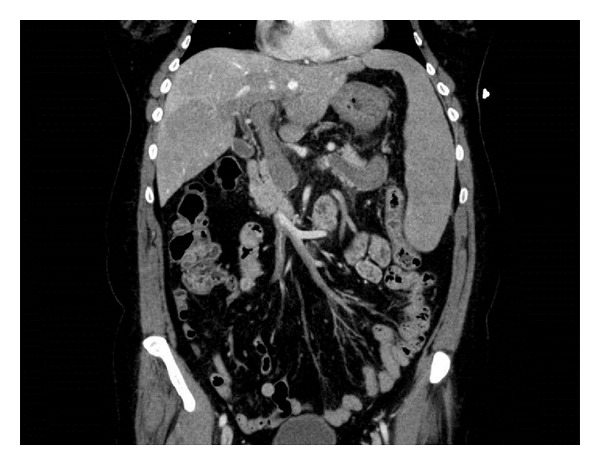
Superior mesenteric thrombosis.

A 59-year-old female with a history of essential thrombocythemia and heterozygous prothrombin gene mutation was hospitalized for abdominal pain. Evaluation revealed portal, superior mesenteric and splenic vein thrombosis, and she was started on warfarin (Figures 1, 2, and 3). She presented two months later with one week of dull epigastric abdominal pain which was worse with movement and food and better with lying down. She also had three days of one to three black and tarry stools daily and progressive fatigue. One week priorly, she underwent esophagogastroduodenoscopy (EGD) to screen for gastric cancer with biopsy of a polypoid mass. The patient had requested the evaluation because of a vague family history of gastric cancer. 

Initial vital signs were remarkable for tachycardia with heart rate of 103, but otherwise benign with a blood pressure of 120/78, respiratory rate of 16, temperature of 98.3 F, and oxygen saturation of 98% on room air. Examination revealed no conjunctival pallor, moist mucosal membranes, and no acute distress. Abdominal examination revealed mild tenderness to palpation of the epigastric region, without guarding, rebound, rigidity, or organomegaly; normoactive bowel sounds; and no stigmata of chronic liver disease.

Initial laboratory evaluation revealed hemoglobin of 10.4 g/dL (she had a normal hemoglobin value of 14.6 g/dL eight weeks prior to presentation), white blood cell count of 10.1 × 10^9^/L, a platelet count of 325 × 10^9^/L, prothrombin time (PT) of 33.4 (11.7−14.2 sec), partial thromboplastin time (PTT) of 41 (24−36 sec), and international normalized ratio (INR) of 3.6 (0.8–1.3). Alanine aminotransferase, aspartate aminotransferase, alkaline phosphatase, total bilirubin, blood urea nitrogen, and creatinine were all within normal limits. 

**Figure 4:**
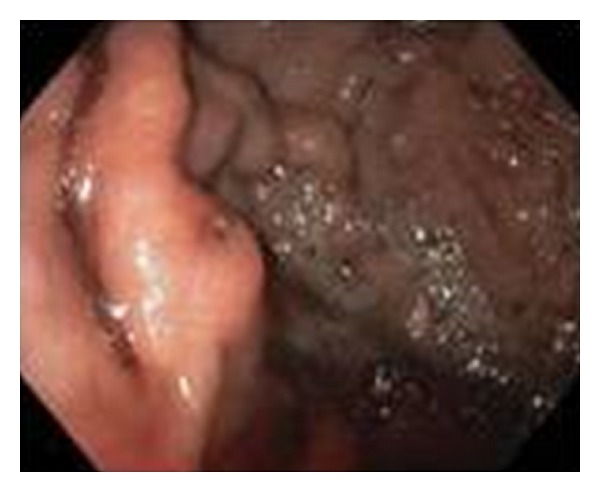
EGD demonstrated a gastric varix with erosion.

She was admitted and started on pantoprazole with an 80 mg IV bolus followed by a maintenance rate of 8 mg/hour. She was typed and crossed for two units of packed red blood cells and received two units of fresh frozen plasma. Repeated CBC the following morning showed that her hemoglobin decreased from 10.4 to 7.1 g/dL, and she was transfused with two units of PRBC and two more units of FFP. INR following the transfusion was 1.7. EGD revealed isolated fundic varices with an erosion over a moderately large gastric varix (Figure 4). Intravenous octreotide at 50 mcg/hr and propranolol 20 mg orally twice a day were started.

**Figure 5:**
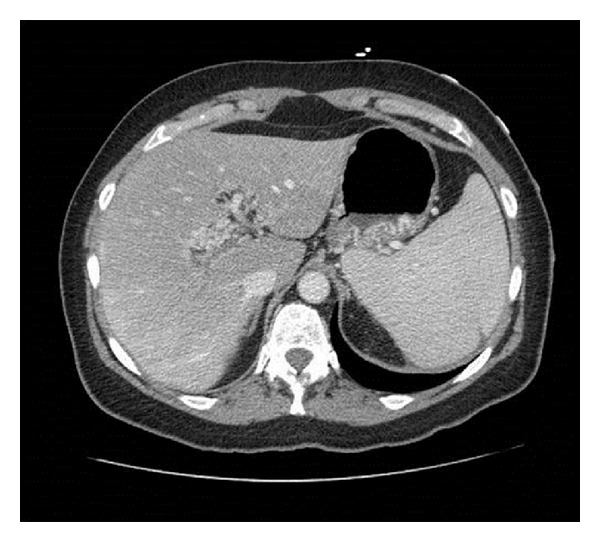
CT demonstrated periportal collateral circulation and gastric varices.

**Figure 6:**
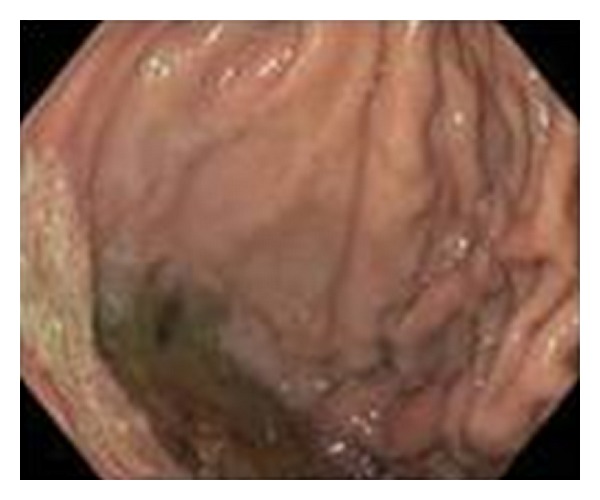
Follow-up EGD demonstrated resolution of varices.

Abdominal computed tomography (CT) showed reduced clot burden within the portal, splenic, and superior mesenteric veins compared to her recent hospitalization; however, she also had new periportal collateral veins and fundic gastric varices (Figure 5). In spite of conservative measures, she continued to bleed with another decrease in hemoglobin to 7.1 g/dL. The patient was transfused one more unit of PRBC and vaccinated for encapsulated organisms, and surgery was consulted. Hand-assisted laparoscopic splenectomy was performed after reviewing all possible options and risks, and benefits were discussed with the patient. Postoperative EGD demonstrated near complete resolution of gastric varices (Figure 6). Twelve months after discharge on warfarin, there has been no reported recurrence of hemorrhage. Repeated abdominal CT imaging one year after discharge showed no significant interval change in splenic, portal, and mesenteric veins thromboses. Also, prominent periportal collateral veins as well as prominent veins near the gastric fundus persisted. 

## 3. Discussion

Gastric varices (GV) are generally divided into those that are a result of splenic vein thrombosis (SVT) and those from portal hypertension (cirrhotic or noncirrhotic). SVT usually develops in the context of acute or chronic pancreatitis, pancreatic pseudocyst, or neoplasm [4–7]. However, GV arising from portal hypertension are more common than from SVT [1]. SVT-associated GV tend to present as multiple varices and are often difficult to manage endoscopically because of bleeding recurrence in alternative short gastric connections [4]. For these patients, splenectomy often resolves the varices. 

An estimated 30% of cirrhotic patients develop variceal bleeding, and of these, approximately 10% to 20% are gastric varices [8, 9]. Gastric variceal bleeding tends to be more severe and to have greater morbidity and mortality than esophageal variceal bleeding [9]. Fundal varices have accounted for up to 80% of bleeding GV in one series [9]. 

Fundal varices often appear as serpiginous, vascular structures or may also present as polypoid masses [4]. Fundal varices may present as an acute, active hemorrhage or incidentally discovered varices. Their polypoid appearance has led to errant biopsy in patients without known liver disease or thrombosis [4]. High-risk GV with recent bleeding or bleeding fundal varices are often difficult to treat. Previous studies have shown a high failure rate for acute control and an early rebleeding rate with sclerotherapy [10]. 

Initial treatment of variceal hemorrhage involves octreotide and balloon tamponade, followed by either surgery or transjugular intrahepatic portosystemic shunt (TIPS). Gastric varices secondary to portal hypertension are often amenable to emergency TIPS for short-term control [11]. In hemorrhage from varices secondary to isolated SVT, splenectomy is the preferred treatment. Splenectomy decompresses the short gastric vessels by decreasing the inflow from the splenic circulation. 

Splenectomy is a known treatment for gastric varices secondary to isolated SVT; however, the presence of multiple thromboses complicates treatment decisions. Our patient was also not a candidate for a shunt procedure. Although the lack of esophageal varices and the transformed portal veins was reassuring, complications of splenectomy in this context may include further thrombosis of the mesenteric system, worsening of the right-sided portal hypertension, and subsequent development of esophageal varices. 

## 4. Conclusion

Gastric variceal bleeding may be caused by portal hypertension or splenic vein thrombosis. In the context of portal hypertension, emergency TIPS is often successful in controlling hemorrhage. Splenectomy is often reserved for patients with isolated splenic vein thrombosis, and in the context of multiple splanchnic and portal thrombosis, treatment is more complicated. We report that splenectomy was a successful treatment for this patient with gastric varices and multivessel extrahepatic thromboses secondary to essential thrombocythemia.
